# Involvement of Perimovement Neural Beta‐Oscillations in Strategic Aiming for Motor Adaptation

**DOI:** 10.1111/ejn.70260

**Published:** 2025-09-26

**Authors:** Matthias Will, Betina Korka, Max‐Philipp Stenner

**Affiliations:** ^1^ Leibniz Institute for Neurobiology Magdeburg Germany; ^2^ Department of Neurology Otto‐von‐Guericke University Magdeburg Germany; ^3^ Zander Labs Munich Germany; ^4^ Center for Behavioral Brain Sciences Magdeburg Germany; ^5^ Center for Intervention and Research on Adaptive and Maladaptive Brain Circuits Underlying Mental Health, Jena‐Magdeburg‐Halle Magdeburg Germany

**Keywords:** beta‐band oscillations, cognitive strategy, explicit adaptation, postmovement beta rebound, premovement beta power event‐related desynchronization, visuomotor rotation

## Abstract

Humans rely on cognitive strategies to adapt upcoming movement in response to past movement error, for example, by strategic reaiming. We show that strategy‐based motor adaptation engages premovement and postmovement neural oscillations in the beta frequency band. We recorded electroencephalography (EEG) while healthy participants (*N* = 27) performed center‐out reaching movements to move a cursor on a screen through a visual target. In some trials (17%), the cursor was unexpectedly rotated relative to the hand. This rotation was either repeated in the next trial, so that participants could reaim their reach in that trial (2× condition), or the rotation was switched off, preventing reaiming (1× condition; within‐subject design). We found a stronger decrease in postmovement beta rebound (PMBR) after the first rotation in the 2× condition, compared to the 1× condition, despite similar movement kinematics. This indicates a role of PMBR in strategic reaiming, and replicates findings from our previous study (Korka et al., 2023). Combining data from the two studies (total *N* = 52), we found that reaiming accuracy was associated with premovement beta power in the second rotated trial, but not with the PMBR decrease at the end of the first rotated trial. Our results indicate that the decrease in PMBR upon movement error signals the need to adjust a cognitive strategy. Such a role may explain how reduced PMBR in Parkinson's disease could impair discovery of cognitive strategies for movement. Premovement beta power, on the other hand, may be involved in the specification of an aiming strategy following erroneous movement.

## Graphical abstract

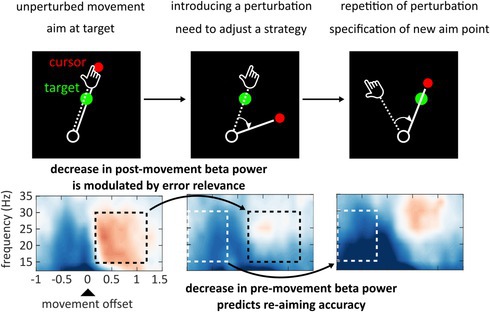

**Abbreviations**
AIC: Akaike information criterion
CD: cerebellar degeneration
EEG: electroencephalography
FIR: finite impulse response
ICA: independent component analysis
LMM: linear mixed model
PD: Parkinson's disease
PMBR: postmovement beta rebound
RT: response time

## 1 Introduction

In order to achieve our goals, we continuously adapt our movements to changes in the environment. Motor adaptation arises from several learning mechanisms. These include implicit adaptation and learning via cognitive strategies, both of which are driven by movement error. A visuomotor rotation, for example, introduces an error between the predicted and the observed visual feedback of movement. This so‐called sensory prediction error drives implicit motor adaptation by adjusting an internal model of the relationship between a motor command and the consequent state change of the body and environment (Mazzoni and Krakauer 2006; Tseng et al. 2007; Wolpert et al. 1995). As a result, movement shifts in a direction opposite to sensory prediction error and thus gradually compensates for the visuomotor rotation. This shift persists as a transient after‐effect once the rotation has been removed, even when a person aims directly at the target. Thus, after‐effects demonstrate that implicit adaptation is highly automatic.

Strategy‐based adaptation, on the other hand, is a deliberate, cognitively more effortful process that aims to reduce target error, that is, any error in hitting the target. To reduce target error, humans can strategically select an alternative course of action, for example, by aiming in the direction opposite to a visuomotor rotation (McDougle and Taylor 2019; Taylor et al. 2014). Several experimental manipulations exist that can dissociate implicit adaptation and strategy‐based learning at a behavioral level (Hadjiosif and Krakauer 2021; Haith et al. 2015; Maresch et al. 2021; Morehead, Taylor, et al. 2016). However, the neural circuitry that gives rise to different motor adaptation mechanisms is not yet well understood.

Motor adaptation is accompanied by modulations of neuronal population signals that can be captured using electroencephalography (EEG) (Reuter et al. 2022). However, the precise information content of these signal modulations with respect to the mental operations involved in motor adaptation is still unclear. Among these signals, movement‐related modulations of oscillations in the beta band (13–30 Hz) have attracted considerable attention (Alayrangues et al. 2019; Jahani et al. 2020; Rilk et al. 2011; Rustighi et al. 2010; Savoie et al. 2018; Spapé and Serrien 2010). Beta power displays prominent modulations around the time of movement (Kilavik et al. 2013). It decreases before and during movement, and rebounds shortly after movement offset (postmovement beta rebound [PMBR]). When sensory feedback for movement is perturbed, for example, by a visuomotor rotation, PMBR is reduced (Palmer et al. 2019; Tan, Jenkinson, et al. 2014). However, it is still a matter of debate how exactly this modulation relates to motor adaptation. For example, Tan et al. (Tan, Wade, et al. 2016) have proposed that the decrease in PMBR mirrors a decrease in confidence in estimates of an internal model. Tan et al. thus suggested a link between PMBR and a key mechanism thought to underlie implicit motor adaptation.

However, given that the study by Tan et al. (Tan, Wade, et al. 2016) employed a large visuomotor rotation that remained constant for many consecutive trials in the critical conditions, it is likely that they emphasized strategy‐based learning (Morehead, Qasim, et al. 2015). Indeed, Torrecillos et al. (Torrecillos et al. 2015) observed a decrease in PMBR even in response to a target jump, an experimental manipulation that does not produce any implicit motor adaptation. They suggested, therefore, a more general role of PMBR in the processing of error saliency, which is compatible with a role for strategy‐based learning (Tsay et al. 2024). In a direct comparison of motor adaptation with versus without strategic reaiming, we have recently shown that the decrease in PMBR in response to a newly introduced visuomotor perturbation depends on whether or not subjects employ a reaiming strategy (Korka et al. 2023). Specifically, the decrease in PMBR is more pronounced when subjects reaim, compared to purely implicit adaptation.

Here, we replicate this finding, using a paradigm that further deconfounds reaiming and the type of perturbation. In our previous study, we compared adaptation to a clamped rotation (or error‐clamp), which renders reaiming futile, with adaptation to a visuomotor rotation, which allows for reaiming. Error‐clamping entails visual feedback that moves always in the same direction relative to the target, irrespective of the direction of hand movement, resulting in a constant error that is beyond the participants' control (Morehead, Taylor, et al. 2016). Participants were fully briefed about the manipulation and instructed to ignore it. While this can effectively prevent reaiming, error‐clamping can also lead to a further distortion in the mapping of hand trajectories to visual feedback, for example, when the hand follows a curved path, while the clamped visual feedback remains always straight.

To avoid any difference in the relation between movement and sensory feedback between conditions, the present study employed the same type of perturbation, that is, a visuomotor rotation, across conditions, and prevented reaiming purely through prior knowledge of the sequence of perturbations. Specifically, participants knew in advance whether a visuomotor rotation that was introduced unexpectedly on a given trial would be repeated in the next trial or not. When they knew that the rotation would be repeated, they could thus reaim their next movement accordingly. We expected a stronger PMBR decrease after a visuomotor rotation when participants knew that the rotation was informative for reaiming the next reach, compared to when the same visuomotor rotation had no relevance for the next movement because it was not repeated.

The second goal of the present study was to reveal whether the responsiveness of PMBR to error predicts a person's success in strategy‐based motor adaptation. Given that PMBR decrease has been associated with error saliency (Torrecillos et al. 2015), and that salient perturbations promote reaiming (Morehead, Qasim, et al. 2015), the degree to which a person's PMBR decreases upon movement error may reflect that person's ability to detect the need for an aiming strategy. This could have important implications for understanding motor adaptation deficits typically observed in Parkinson's disease (PD) and cerebellar degeneration (CD). Both patient groups have greater difficulty discovering a reaiming strategy than healthy controls (Butcher et al. 2017; Tsay, Najafi, et al. 2022; Wong et al. 2019). Interestingly, the PMBR is lower in PD and CD compared to healthy controls (Pfurtscheller et al. 1998; Tamás et al. 2003; Aoh et al. 2019; Visani et al. 2020). This low PMBR, even for unperturbed movements, may limit the PMBR's responsiveness to error when a perturbation is introduced (Klimpke et al. 2020) and could thus hamper discovery of a strategy for motor adaptation.

Here, we explored the relationship between individual PMBR and individual reaiming accuracy in healthy individuals. To increase power, we took advantage of the highly similar design of the reaiming condition in the present study and our previous study (Korka et al. 2023). This high similarity allowed us to collapse data from the present study with data from our previous study, yielding a total sample size of 52 participants.

A cognitive strategy entails updating a motor plan. This update likely occurs while people specify a new aim point, that is, during preparation for the next movement following an error. Indeed, limiting movement preparation time hampers reaiming success (Haith et al. 2015; McDougle and Taylor 2019). Movement preparation and execution are characterized by a decrease in beta power in the EEG (Kilner et al. 2005; Stancák and Pfurtscheller 1995; for review, see Kilavik et al. 2013). Like the PMBR, premovement beta oscillations undergo systematic modulation during motor adaptation (Darch et al. 2020; Jahani et al. 2020). This modulation could thus reflect specification of a strategy. In a post hoc analysis, we therefore explored whether individual differences in the modulation of premovement beta power during adaptation predict reaiming accuracy.

## 2 Materials and Methods

### 2.1 Participants

Thirty healthy right‐handed volunteers completed the experiment. Handedness was determined via the Edinburgh Handedness Inventory (Oldfield 1971). Three participants did not follow reaiming instructions, evident in a high number of rejected trials (see “Analysis of kinematic data” for rejection criteria), and were thus excluded. The final sample size of 27 participants included 17 females and 10 males. Participants were, on average, 28.4 ± 7.6 years old (mean ± standard deviation). The sample size was chosen to closely match the sample size in our previous study (Korka et al. 2023; *N* = 26, 14 female, mean age 23.8 ± 6.8 years). This sample size ensured more than 90% power to detect an effect of reaiming on the dynamics of PMBR amplitude with an effect size that was at least as large as in our previous study (*d* = 0.87). One participant's dataset from the earlier study could not be retrieved correctly. As a result, combining data from both studies yielded a total of 52 participants. Participants gave written informed consent, and the study was approved by the ethics committee of the Medical Faculty of the University Magdeburg and conducted in accordance with the Declaration of Helsinki. Participants received monetary compensation.

### 2.2 Apparatus

**FIGURE 1:**
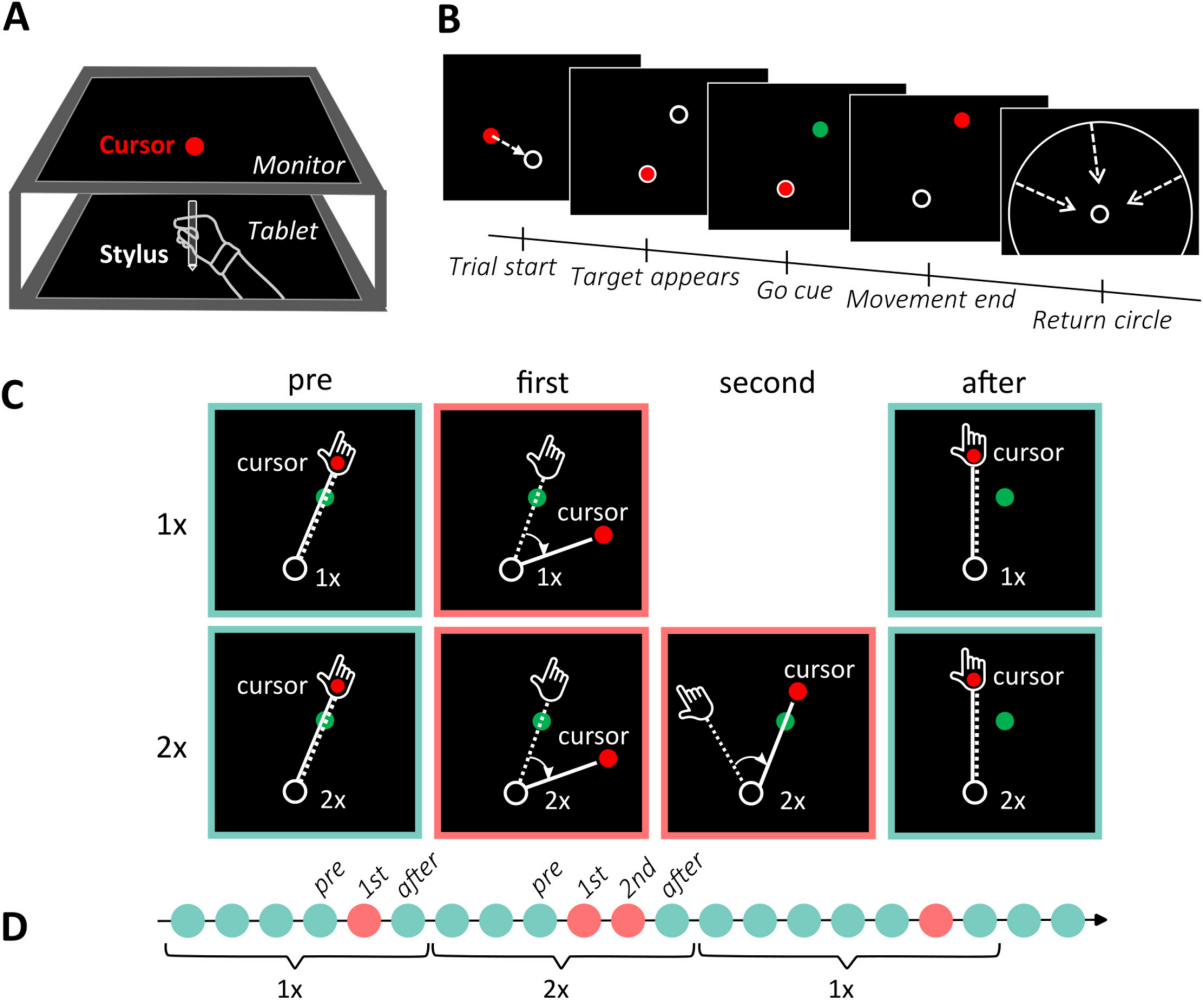
Set‐up and task. (A) The experimental set‐up consisted of a tablet, on which participants performed center‐out reaching movements holding a stylus, and a monitor mounted above the tablet, on which online visual feedback was displayed. (B) Participants moved the cursor (red dot) to the home position (white circle) at the beginning of each trial. After holding the stylus in place for 2000–2500 ms, the target appeared (white circle). Once the target turned green (1500 ms after target onset), participants could initiate a reaching movement to slice the cursor through the target. After movement offset, participants held their hand in the endpoint position for a short duration (1600–2100 ms). A white circle, whose radius corresponded to the radial distance of the hand from the home position, guided the return movement. (C) Cursor and hand position were identical on most trials (cyan frame). On some trials, the cursor trajectory was rotated relative to hand movement (red frame). In each cycle, there could be a single trial with a rotation (1× condition, upper row), or two consecutive trials with identical rotation (2× condition, lower row). In both conditions, the first trial with a rotation was not predictable, so that participants could not anticipate the rotation, and the cursor missed the target on the first rotated trial. Participants were instructed to compensate for the rotation in the second trial with a rotation in the 2× condition by reaiming their reach in the opposite direction of the rotation. Following the second rotated trial (2× condition) or single rotated trial (1× condition), cursor and hand position were again aligned, and participants again aimed directly at the target (after‐trial). We expected that participants would implicitly adapt their movements to the visuomotor rotation in both conditions and consequently show an after‐effect in the after‐trial, evident in slightly missing the target in the direction opposite to the rotation. (D) Conditions alternated after every cycle.

Participants were sitting in a quiet, dimly lit, electrically shielded chamber (Industrial Acoustics Company). Both in this study and our previous study (Korka et al. 2023), participants moved a stylus held in their right hand across a graphics tablet (Wacom Intuos Pro Large, Kazo, Japan; sampling rate of 200 Hz, active area of 311 × 216 mm). The stylus tip was continuously in contact with the tablet, which recorded its position. An LCD screen (refresh rate 60 Hz) mounted horizontally above the tablet occluded vision of the hand and provided visual feedback (see Figure 1A).

### 2.3 Task and Procedure

Participants performed center‐out reaching movements with their dominant right hand in a horizontal plane to make a cursor on the screen “slice” through a visual target. Stimuli and events in individual trials were identical to our previous study (Korka et al. 2023). Trials started with the hand holding the stylus still in the home position (white circle, outer diameter of 8.5 mm, see Figure 1B). After a variable time interval between 2000 and 2500 ms (uniform random distribution), the target (white circle, outer diameter of 6 mm) appeared 2 cm away from the home position at an angle of 45° clockwise relative to the “straight ahead” direction. Participants were instructed to initiate the movement once the target turned green (1500 ms after target onset), with no time limit for movement initiation after that. Movement initiation was defined as the time point when the tip of the stylus left the home position. Throughout the movement, participants saw a cursor (red dot, diameter 5 mm) as visual feedback of their movement. Participants had to cross the target radius within a time window of 50–160 ms after movement initiation. If they crossed the target distance earlier than 50 ms, or later than 160 ms, or if they initiated their movement before the target turned green, they saw a corresponding error message on the screen (“TOO FAST,” “TOO SLOW,” or “TOO EARLY,” respectively). Movement offset was defined as the point in time when the cursor did not change position for at least two vertical frame refreshes, that is, for 33.3 ms. After stopping their movement behind the target, participants had to hold the stylus in place (in the endpoint position) for at least 1600–2100 ms (uniform random distribution). If participants moved their hand more than 5 mm away from the endpoint position during this period, they received an error message (“HOLD STILL”). They then moved their hand back to the home position, guided by a circle whose radius corresponded to the distance of the hand from the home position. This prevented feedback about the angular position of their hand during the return movement, while providing information about the radial distance from the home position. The trial ended when the hand reached the home position (see Figure 1B).

The experiment consisted of 16 blocks of 72 trials each, which participants completed over the course of 2 days of testing (8 blocks per day). Trials in each block were organized in 12 cycles of different lengths (at least 4 trials/cycle). In most trials (54 per block), cursor and hand position were aligned (unrotated trials). In the other, pseudo‐randomly interspersed trials, the movement of the cursor was rotated relative to the movement of the hand (rotated trials, 18 per block). There were two conditions: each cycle contained either a single trial with a rotation (1× condition; 50% of cycles) or two consecutive trials with a rotation (2× condition; see Figure 1C). Throughout each cycle, participants saw the characters “1×” or “2×” on the screen (below the home position), indicating the current condition. Thus, they knew beforehand whether any rotation they encountered during a given cycle would be repeated once in the subsequent trial (2×) or not (1×). To minimize the possibility that participants used a single, fixed strategy throughout the experiment, rotations differed in magnitude (30°, 37.5°, or 45°) and direction (clockwise or counterclockwise) across cycles. Each of the three rotation magnitudes and two rotation directions occurred in an equal number of cycles per block and equally often in cycles of each condition. Importantly, in the 2× condition, the two consecutive rotated trials had the same rotation magnitude and direction. This allowed participants to reaim their reach in the second rotated trial, based on the rotation experienced in the first rotated trial, so that the cursor would slice through the target. Because of variable cycle length, participants could not form any reliable prediction about when a rotation was going to occur for the first time in a cycle. We therefore expected participants to follow the default instruction to aim their movement directly at the target, except in the second rotated trial in the 2× condition. After the last rotated trial in each cycle, there was a further unrotated trial, in which participants were instructed to aim directly at the target again, allowing us to measure after‐effects of implicit adaptation. Following that trial, the text below the home position changed (from “1×” to “2×,” or back), prompting the change in condition for the upcoming cycle (see Figure 1D).

Similarly to the present study, participants in our previous study (Korka et al. 2023) had to ignore or compensate for a visuomotor perturbation. There were two conditions with different types of visuomotor perturbations. In contrast to the present study, the *Ignore* condition used movement direction‐invariant (or clamped) visual feedback, rendering any reaiming useless (Morehead, Taylor, et al. 2016). The other condition (*Compensate*) was virtually identical to the 2× condition in the present study, with the only exception that participants completed entire blocks of trials of the *Compensate* condition, rather than shorter cycles of trials (for details, see Korka et al. 2023). For the analysis of the relationship between reaiming accuracy and beta activity in the present manuscript, we included only data from the *Compensate* condition.

### 2.4 Analysis of Kinematic Data

Data analysis (behavioral and EEG) for the present study was similar to our previous study (Korka et al. 2023). The primary kinematic variable of interest in this study was movement direction relative to the target at the point of maximum velocity. Movement direction for rotated trials and corresponding after‐effect trials was defined as positive for the direction opposite to the rotation. We also computed response time (RT), movement time, movement extent, and movement curvature. Response time was defined as the duration between the Go signal (the target turning green) and movement initiation. Note that participants could plan a movement earlier than the Go signal because the target was displayed before, and target location remained constant throughout the experiment. Movement curvature was computed as the maximum perpendicular distance between a movement trajectory and a straight line from the home position to the movement endpoint, relative to movement extent (Atkeson and Hollerbach 1985).

We excluded all trials in which participants received an error message (“TOO SLOW,” “TOO FAST,” “TOO EARLY,” and “HOLD STILL”), as well as trials that followed trials with one of the former two error messages. Finally, we excluded cycles in which participants did not follow the aiming instructions, that is, cycles in the 2× condition in which movement angle in the second rotated trial was < 15° away from the target, and cycles in the 1× condition in which movement angle in the trial following the single rotated trial was > 15° away from the target, that is, we used the same cutoff of movement direction as in our previous study (Korka et al. 2023).

We report results for the pre‐trial (trial before the first rotation in each cycle), first rotated trial, second rotated trial (present only in the 2× condition), and after‐trial (first unrotated trial after the rotation in each cycle, see Figure 1D).

### 2.5 EEG Data Recording

In each session, EEG was recorded using 35 Ag–AgCl passive electrodes connected to a BrainAmp amplifier, with data acquisition handled by the Vision Recorder software (Brain Products). The sampling rate was set at 500 Hz. Two electrodes were positioned on the mastoids, with the right mastoid serving as the online reference. The ground electrode was placed at the Fpz location. Three electrodes were used for recording EOG activity: two were placed on the outer canthi of the left and right eyes, and one below the left eye. The remaining 29 electrodes (Fp1/2, F3/4, F7/8, FC1/2, C3/4, CP1/2, T7/8, P3/4, P7/8, PO3/4, PO7/8, O9/10, Fz, Cz, Pz, Oz, Iz) were mounted in an elastic cap (EasyCap) according to the extended international 10–20 system. Before beginning data recording, we ensured that all electrode impedances were below 10 kΩ. The raw EEG data are available online under 10.5281/zenodo.14673450 and 10.5281/zenodo.14674428 (together with the kinematic data 10.5281/zenodo.14671785).

### 2.6 Analysis of EEG Data

The signals of interest for EEG analysis were the PMBR and premovement beta power. EEG preprocessing was performed in MATLAB R2020b (Mathworks) using the FieldTrip toolbox (Oostenveld et al. 2011). First, the continuous raw data was filtered using a 100 Hz low‐pass and a 0.1‐Hz high‐pass windowed sinc finite impulse response (FIR) filter (Hamming window, filter order 66‐low‐pass and 8250‐high‐pass). Next, the continuous data set was epoched, with epochs starting 2 s before target onset and ending 2 s after beginning of the return movement. We removed epochs with exceptionally high amplitude fluctuations. Specifically, for each epoch we computed the “robust standard deviation” (i.e., 0.7413 times the interquartile‐range; Bigdely‐Shamlo et al. 2015) of EEG amplitudes across time for each channel, and then calculated the median robust standard deviation across all channels. Epochs were rejected using a thresholding approach based on the “robust *z* score” (Bigdely‐Shamlo et al. 2015) of the obtained epoch distribution (robust *z* > 6). This threshold was chosen to strike a balance between maintaining enough data and rejecting clear outliers, which was confirmed by visual inspection. We removed 11.1 trials on average (range: 0–37). Similarly, we computed the robust standard deviation after concatenating all trials, for each channel separately. Noisy channels were identified using the robust *z* score of the channel distribution (robust *z* > 3) and removed. On average we removed 1.1 channels (range: 0–4). Rejected channels were replaced with a weighted average of all neighbors. Following this, we ran an ICA on a copy of the data. This copy was processed in a similar way as described above with the difference that the applied high‐pass filter used a higher cutoff frequency of 1 Hz (windowed sinc FIR filter, filter order 826, same low‐pass filter as described before) to optimize identification of eye movement and muscle artefacts via ICA. We also used a more rigorous trial rejection threshold for this copy of the dataset (robust *z* > 3) to avoid any bias to the ICA due to artifactual trials. Components related to eye movements and muscle artefacts were identified via visual inspection and the obtained mixing and unmixing matrices were applied to the original dataset, removing the identified eye movement components (on average 2.7 components, range: 1–4). As the final step in the preprocessing pipeline, we re‐referenced to a common average across all electrodes (not including EOG).

We performed a time–frequency analysis (*ft_freqanalysis* function in FieldTrip with “mtmconvol” method and a 400 ms Hanning taper) from which we obtained spectral power for frequencies from 2.5 to 40 Hz (in steps of 2.5 Hz) and time points from −3200 to 2200 ms relative to movement offset (in steps of 50 ms). A shared baseline for the 1× and 2× conditions was defined as the spectral power during the intertrial interval (period of −1800 to −200 ms relative to target onset, spectral power averaged over time) before unrotated trials (except pre‐trial and after‐trial) from both conditions. Spectral power was averaged across trials, separately for each condition, that is pre‐trials, first and second rotation, and after‐trials, each for the 1× condition and 2× condition.

**FIGURE 2:**
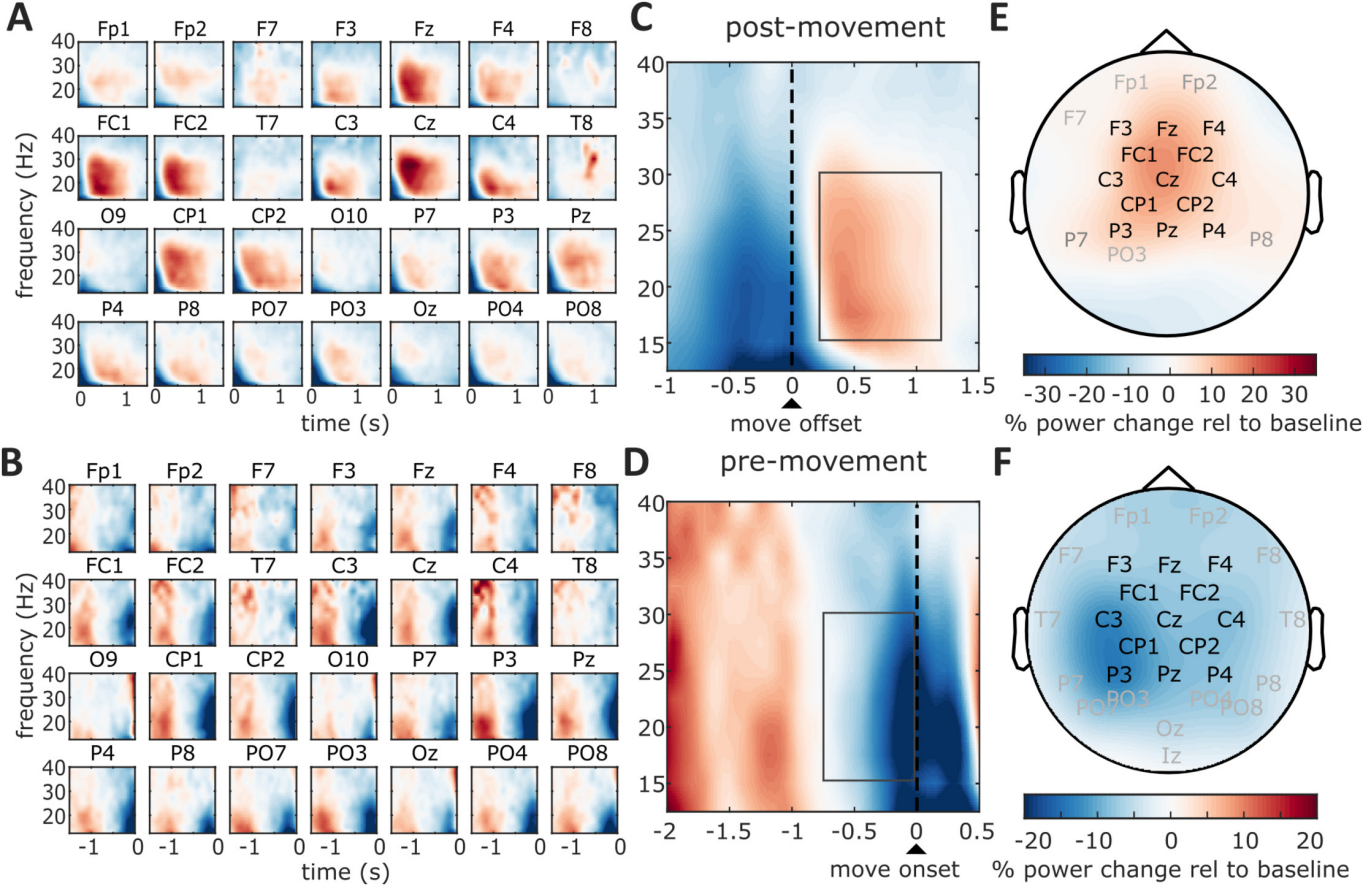
Channels and time–frequency windows of interest. (A and B) Channel selection for postmovement (upper row) and premovement (lower row) beta power analysis was based on visual inspection of individual channels showing prominent activity in the respective time window. (C and D) Power change relative to baseline, averaged across the channels of interest shown in panel E and F. The black rectangles indicate the premovement and postmovement time–frequency windows of interest. (E and F) Channels of interest selected based on visual inspection (black labels) and cluster‐permutation test (grey labels). The same visually identified channels were used for the premovement and postmovement beta power analysis. The color codes for power change, in the time–frequency window of interest, relative to baseline (1800–200 ms before target onset, same frequency window).

We identified channels of interest for PMBR analysis. Importantly, channel identification was performed only on unrotated trials (except pre‐trial and after‐trial), that is, trials that are not included in the statistical PMBR analysis, which is therefore unbiased by this selection step. We defined channels of interest based on visual inspection by selecting channels that showed a prominent PMBR, evident as an increase in spectral power in the beta‐frequency range after movement offset relative to baseline (Figure 2A). Channels identified in this way included bilateral fronto‐central and centro‐parietal channels, in line with previous EEG studies of the PMBR (e.g., Tan, Jenkinson, et al. 2014). We refined the range for the time and frequency of interest by inspecting spectral power averaged over the selected channels and identified a time–frequency window of interest that ranged from 15 to 30 Hz, and from 200 to 1200 ms after movement offset (Figure 2C). We verified this selection of channels and time–frequency window of interest by comparison with a positive cluster obtained via FieldTrip's cluster‐based permutation test (*p* = 0.049), which compared beta‐power in a broader postmovement time–frequency window (12.5–40 Hz, 100–1800 ms) and across all channels to baseline power (as defined above). This test revealed a time and frequency range that was similar to the selection by visual inspection and a slightly larger number of channels that displayed an increase in postmovement beta power, which included more frontal and parietal channels in addition to the channels of interest identified via visual inspection (channel labels in grey and black font, respectively, in Figure 2E). Subsequent PMBR analyses were based on the selection of channels of interest by visual inspection, which matched better with the topography found in our previous study (Korka et al. 2023). However, results are qualitatively identical when using the larger selection of channels of interest. Finally, we obtained a single value as a measure for the PMBR for pre‐rotation and first rotated trial and 1× and 2× condition by averaging the change in spectral power relative to baseline across the channels and time–frequency window of interest.

The identification of channels and the time–frequency window for the premovement period followed the same approach as for PMBR. Channels of interest were first defined through visual inspection, selecting those showing the largest premovement beta desynchronization during unperturbed reaches. Channels with a prominent PMBR (black font in Figure 2E) also displayed a prominent premovement event‐related beta desynchronization, in agreement with previous studies of premovement beta desynchronization (Jahani et al. 2020). A cluster‐based permutation test comparing beta power between unperturbed trials and baseline within a broader time–frequency range (12.5 to 40 Hz, −1000 to 0 ms) identified a premovement beta desynchronization in all channels (except O9 and O10; *p* = 0.002; channel labels in grey and black font in Figure 2F). Such widespread patterns likely reflect the conflation of neighboring channels, a known limitation of clustering methods due to inherent spatiotemporal correlations in EEG signals arising from volume conduction. Given that the previous literature points to a central generation of the movement‐related desynchronization of beta power (Pfurtscheller and Lopes da Silva 1999), our primary analysis was based on the channels shown in black font in Figure 2F (i.e., the same channels as for the PMBR analysis). However, using the broader definition of channels of interest (from the cluster‐based permutation test) did not change any statistical inference.

We refined the time–frequency window by averaging premovement beta power across these channels. For unperturbed trials, beta desynchronization was most prominent during the final 400 ms before movement onset and within a frequency range of 15–30 Hz (Figure 2D). Anticipating that movement planning would begin shortly after target onset, we extended this window to 800 ms before movement onset. Note that target onset occurred 1500 ms before the go signal (the earliest possible movement onset); however, a prominent beta synchronization was still present at that time, possibly reflecting beta power induced by the return movement at the end of the preceding trial. For this reason, we analyzed only part of the premovement period.

### 2.7 Linear Mixed Model

After we confirmed that PMBR was modulated by use of an aiming strategy, we asked if individual differences in the decrease in PMBR when a rotation was first introduced, or individual differences in the decrease in premovement beta while reaiming, could predict reaiming accuracy. To this end, we performed linear mixed effects analyses of the relationship between reaiming performance, computed as unsigned target error on individual second rotated trials, and beta power. In different linear mixed models (LMMs), we included as fixed effects the PMBR decrease from the pre‐trial to the first rotated trial, or the premovement beta decrease on the second compared to the first rotated trial. Given its relevance for movement preparation, we included RT in the second rotated trial as a fixed effect in the premovement model. As a random effect, both LMMs included intercepts for individual subjects. *p* values were obtained from likelihood ratio tests of the full model including PMBR or premovement beta decrease as fixed effects against a reduced (null) model without the respective fixed effect. For these analyses, we included data from a reaiming condition from our previous study, which was virtually identical to the 2× condition of the present study. The only difference between both datasets is that the previous study used a blocked design, while conditions were interleaved in the present study. A total of 52 individuals contributed data (3551 trials in total for PMBR, 1671 from the present and 1880 from our previous study and 3903 trials in total for premovement beta, 1993 from the present and 1910 from our previous study) for the linear mixed‐effects analyses.

### 2.8 Statistics

We report mean values and standard deviation, or median and interquartile range (IQR), when a Shapiro–Wilk test indicated a violation of the assumption of normality. Movement kinematics between conditions were compared using paired *t* tests. When the assumption of normality was violated, we used a corresponding nonparametric test. Bayesian *t* tests using a Cauchy prior centered at 0 with a scale parameter of 0.707 for the effect size (δ) were used to test for evidence in favor of the null hypothesis. Repeated‐measures analyses of variance (ANOVAs) were used to test for differences in movement direction between rotation magnitudes (30°, 37.5°, 45°) and differences in PMBR between conditions (1× and 2×) and trial types (pre‐trial and first rotated trial). Premovement beta power between the first and the second rotated trial was compared using a paired *t* test. Statistics were computed in MATLAB R2020b and JASP 0.14.0.0 (JASP Team 2020). For the LMMs, we employed the lme4 package (Bates et al. 2015) in R (R Core Team 2024). We report Akaike information criterion (AIC) as a measure of relative model quality.

## 3 Results

Our primary hypothesis was that the decrease in PMBR during visuomotor adaptation would be modulated by the behavioral relevance of movement errors, that is, the error between the rotated cursor and target (target error). We expected a more prominent reduction in PMBR on the first rotated trial, relative to the pre‐trial, when the target error was relevant for the next movement, that is, when it was informative for devising a reaiming strategy (2× condition), compared to when it was not relevant for the next movement, that is, when there was no deliberate change in behavior following the first rotated trial (1× condition).

### 3.1 Kinematic Evidence of Successful Reaiming

We first verified that there were no condition differences in kinematics in the first rotated trial that could confound the EEG analysis. There were no significant differences in RT (1×: 0.55 ± 0.15 s; 2×: 0.56 ± 0.15 s; mean ± SD), movement time (1×: 0.31 ± 0.04 s, 2×: 0.31 ± 0.04 s), movement extent (1×: 4.00 ± 0.98 cm, 2×: 4.00 ± 1.01 cm), maximum movement velocity (1×: 24.72 ± 4.52 cm/s, 2×: 24.67 ± 4.30 cm/s), and movement curvature (linearity index, 1×: 0.09 ± 0.03, 2×: 0.09 ± 0.03) between the 1× and 2× conditions (all *t*(26) < 0.89, all *p* > 0.38). Bayesian paired *t* tests provided moderate evidence that kinematic parameters were indeed similar between conditions (all BF_10_ < 0.3).

**FIGURE 3:**
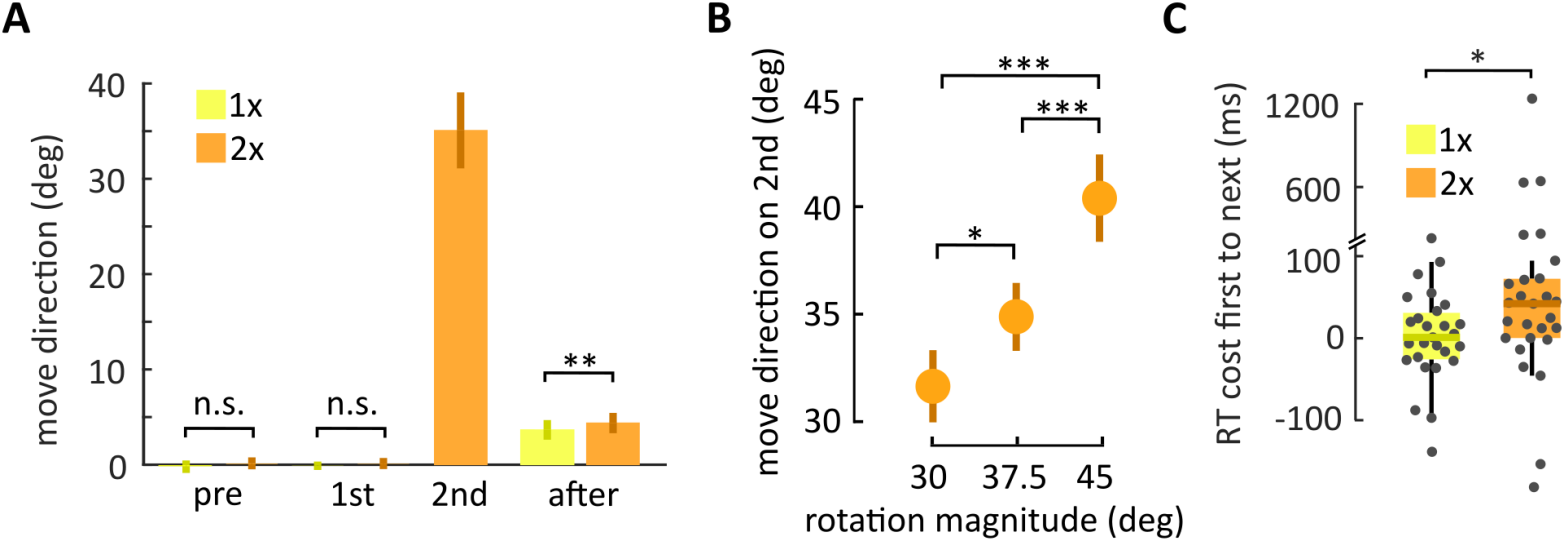
Behavioral results. (A) Mean movement direction (bars, vertical bold lines indicate standard deviation) across different trials. The y‐axis shows movement direction relative to the target. Positive values on the y‐axis indicate movement angles opposite to the rotation that participants experienced in the first rotated trial (and, in the 2× condition, in the second rotated trial). Participants moved their hand to the target during the pre‐trial and first rotated trial in the 1× (yellow) and 2× condition (orange). When a second, identical rotation followed the first rotation, that is, in the 2× condition, participants compensated for the angular deviation by aiming in the opposite direction. On the trial after the rotation, participants were biased in the direction opposite to the previously experienced rotation due to implicit adaptation. (B) Aiming direction in the second rotated trial was selected according to the rotation magnitude (dots indicate mean, vertical bold lines indicate standard deviation). (C) Reaiming was associated with a higher RT cost compared to maintaining aim directly at the target (boxplots, dots represent individual participants).

Next, we examined movement direction across different trial types and conditions. Across the 1× and 2× condition, participants were similarly accurate in slicing through the target during the pre‐trial (1×: −0.21 ± 0.66°, 2×: 0.14 ± 0.62°; *W* = 120, *p* = 0.10, BF_10_ = 1.02) and first rotated trial (1×: −0.11 ± 0.44°, 2×: 0.12 ± 0.58°; *W* = 143, *p* = 0.28, BF_10_ = 0.53; Figure 3A). In the 2× condition, participants compensated for the rotation in the second rotated trial by aiming in the opposite direction, to a degree that was sensitive to the magnitude of rotation (Figure 3B). A rANOVA revealed a main effect of rotation magnitude (30°, 37.5°, 45°) on movement direction (31.66 ± 4.94°, 34.87 ± 4.83°, 40.40 ± 7.00°, respectively; *F*(2,52) = 26.3, *p* < 0.001, $\eta^{2}$= 0.50). Post hoc tests showed that movement direction differed between all rotation magnitudes (30° vs. 37.5°: *t*(26) = 2.64, *p* = 0.011, *d* = 0.57; 30° vs. 45°: *t*(26) = 7.17, *p* < 0.001, *d* = 1.54; 37.5° vs. 45°: *t*(26) = 4.53, *p* < 0.001, *d* = 0.97; all Holm corrected).

A 2 × 2 rANOVA with RTs as dependent variable indicated a significant effect of condition (1× and 2×; *F*(1,26) = 4.65, *p* = 0.040, $\eta^{2}$= 0.05), and a trend for an effect of trial type (first rotated trial and next trial, i.e., after‐trial in 1× condition and second rotated trial in 2× condition; *F*(1,26) = 3.96, *p* = 0.056), as well as an interaction between both factors (*F*(1,26) = 4.80, *p* = 0.038, $\eta^{2}$= 0.04; Figure 3C). RTs increased from the first rotated trial to the next trial in the 2× condition (by 42.0 ± 72.0 ms; median ± IQR; *W* = 64, *p* = 0.048, *r* = 0.61; Holm correction), but not the 1× condition (0.8 ± 53.7 ms; *t*(26) = 0.14, *p* = 1; Holm correction; BF_10_ = 0.22). On after‐trials, movement direction showed a bias in the opposite direction of the rotation as evidence for implicit adaptation (Figure 3A, right). A 2 × 3 rANOVA with the after‐effect as dependent variable, and with the within‐subject factors condition (1× and 2×) and rotation magnitude (30°, 37.5°, 45°; on the previous trial) showed a main effect of condition (1×: 3.66° ± 1.03°, 2×: 4.37° ± 1.07°, *F*(1,26) = 11.51, *p* = 0.002, $\eta^{2}$= 0.14). There was no effect of rotation magnitude (*F*(2,52) = 1.35, *p* = 0.27), nor an interaction between both factors (*F*(2,52) = 0.55, *p* = 0.58).

### 3.2 PMBR Is Modulated by Strategy‐Based Motor Adaptation

**FIGURE 4:**
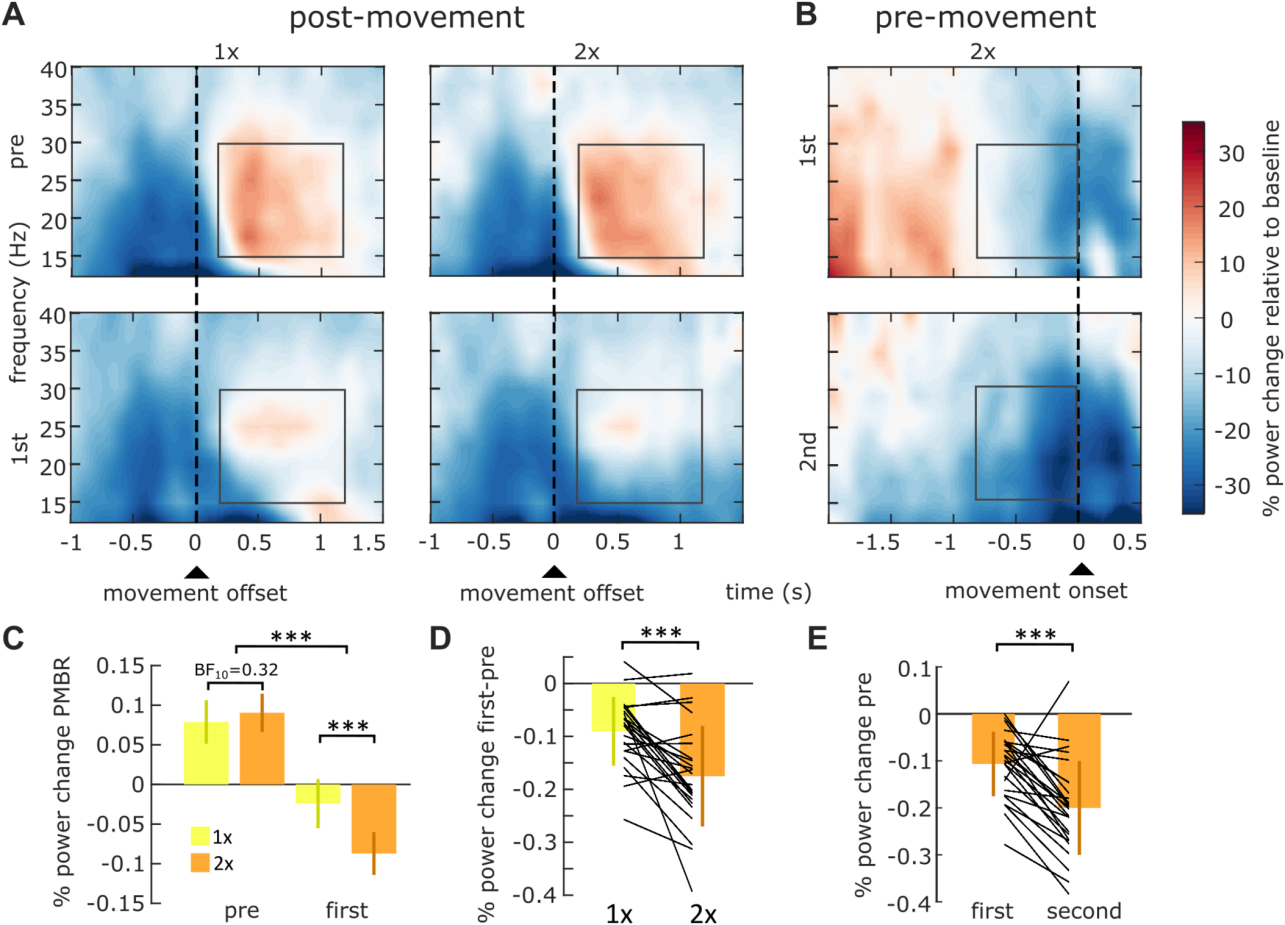
Time–frequency resolved power for the postmovement (A) and premovement time–frequency windows (B), averaged across channels of interest (Figure 2E and F), separately for different trial types (rows) and conditions (columns). (C) PMBR diminished from the trial before (left bars) to the trial when a visuomotor rotation was first introduced (right bars) in the 1× (yellow) and 2× condition (orange). Colored vertical lines indicate ± SEM (D) The PMBR decrease was more pronounced when participants knew that the rotation would be repeated on the following trial, and they were instructed to reaim on the second rotated trial (2× condition, right bar), compared to when they knew that the rotation would not be continued and they would not have to reaim (1× condition, left bar). Black lines indicate individual participants. Colored vertical lines indicate ±STD. (E) Premovement beta power decreased from the first rotated trial to the second rotated trial, when participants prepared to compensate for the perturbation. Black lines indicate individual participants. Colored vertical lines indicate ±STD.

Next, we examined PMBR across different trial types and conditions (Figure 4A). By visual inspection, there was a prominent PMBR on the pre‐trial, which decreased strongly in both conditions when a rotation was introduced. PMBR was slightly decreased on the second rotated trial compared to the pre‐trial. After the rotation was turned off, PMBR was similar to the pre‐trial again in both conditions (Supplementary Figure S1). A formal statistical analysis of PMBR (average across channels of interest, and the time–frequency window of interest) was based on a 2 × 2 rANOVA with the within‐subject factors condition (1× and 2×) and trial type (pre‐trial and first rotated trial), thus only including trials that were matched in kinematics. This revealed a significant main effect of trial type (*F*(1,26) = 115.50, *p* < 0.001, $\eta^{2}$= 0.63; Figure 4C), a significant main effect of condition (*F*(1,26) = 5.20, *p* = 0.03, $\eta^{2}$= 0.02), and, importantly, a significant interaction between both factors (*F*(1,26) = 20.48, *p* < 0.001, $\eta^{2}$= 0.05; Figure 4D). Post hoc tests revealed that there was no significant difference in PMBR between both conditions in the pre‐trial (1×: 5.8 ± 11.9%, 2×: 7.0 ± 10.4%, *t*(26) = 0.96, *p* = 0.33, Holm correction, BF_10_ = 0.32), while there was a significant difference in the first rotated trial (1×: −3.0 ± 14.1%, 2×: −8.6 ± 12.5%, *t*(26) = 4.58, *p* < 0.001, Holm correction, *d* = 0.45; Figure 4C), replicating the key finding of our previous study (Korka et al. 2023).

### 3.3 Interindividual Differences in Premovement Beta, but Not PMBR, Predict Interindividual Differences in the Accuracy of Reaiming

After confirming that PMBR was influenced by the use of an aiming strategy, we investigated whether the reduction in PMBR from the pre‐trial to the first rotated trial predicts individual differences in reaiming accuracy. To explore this, we performed a linear mixed effect analysis of the relationship between reaiming performance, computed as the unsigned error on the second rotated trials, and the corresponding PMBR decrease from pre‐trial to first rotated trial. We used the PMBR decrease as a fixed effect and included intercepts for individual participants as random effects in an LMM. This analysis also included data from a reaiming condition in our previous study, which was virtually identical to the 2× condition in this experiment. Comparison with a model that did not include PMBR decrease showed no significant difference (AIC full model = 23,638, AIC reduced model = 23,636, χ^2^(1) = 0.02, *p* = 0.88). To rule out the possibility that the analysis was biased by our rejection of trials with poor reaiming (movement angle < 15°), which resulted in low unsigned error (on average 8.86 ± 6.64° across all included trials), we repeated the analysis after including trials in which reaiming was poor (average unsigned error: 10.87 ± 8.98°). However, even in this control analysis, the decrease in PMBR was not predictive of reaiming accuracy (AIC full model = 271,115, AIC reduced model = 271,114, χ^2^(1) = 0.25, *p* = 0.62).

We investigated whether beta activity in the premovement time window—associated with movement preparation and potentially critical for reaiming accuracy—could account for variations in reaiming performance. In the 2 × condition, premovement beta desynchronization began earlier and was stronger on the second compared to the first rotated trial (Figure 4B), resulting in a significant power change within the specified time–frequency window from the first (−10.7 ± 6.9%) to the second rotated trial (−20.0 ± 10.0%; *t*(26) = 5.39, *p* < 0.001; Figure 4E). We then examined whether the magnitude of this decrease from the first to the second rotated trial was related to reaiming accuracy, using a similar analysis as for PMBR. An LMM including RT and premovement beta decrease as fixed effects predicted unsigned movement error significantly better than the reduced model that excluded premovement beta decrease as a fixed effect (AIC full model = 27,811, AIC reduced model = 27,818, χ^2^(1) = 9.17, *p* = 0.002), demonstrating a significant effect of premovement beta decrease over and above RT. The model predicted a 0.42° decrease in unsigned error for a premovement beta decrease of 31% relative to baseline, corresponding to one standard deviation of premovement beta decrease across all reaiming trials, while the standard deviation for unsigned error was 8.96°. Thus, the model predicted for roughly 5% of the variability in reaiming accuracy.

## 4 Discussion

We have recently shown that the decrease in PMBR during motor adaptation is amplified when participants are instructed to compensate for movement errors by using reaiming strategies (Korka et al. 2023). However, it has remained unclear to what extent differences in PMBR between participants can explain differences in their ability to accurately compensate for systematic movement errors. Understanding this relationship is crucial for elucidating the role of reduced PMBR in clinical disorders, for example, observed in PD (Pfurtscheller et al. 1998; Tamás et al. 2003) and CD (Aoh et al. 2019; Visani et al. 2020), for deficits in motor adaptation. Here, we replicate the attenuating effect of strategy use on PMBR in a paradigm that avoids potential confounds introduced by different types of perturbation in our previous study. This replication also allowed us to collapse data from the present and previous study to examine the relation between interindividual differences in reaiming accuracy and PMBR or premovement beta in a relatively large cohort of healthy subjects. Surprisingly, while PMBR shows a clear association with reaiming at the group level, it does not predict individual reaiming accuracy. However, reaiming performance was predicted by premovement beta power. We discuss potential reasons for these findings, together with potential future directions.

### 4.1 What Mental Process Does the Modulation of the PMBR Decrease Relate to?

We found that PMBR decreased in response to a visuomotor rotation, an observation shared by multiple studies in the context of motor adaptation (Korka et al. 2023; Tan, Wade, et al. 2016; Torrecillos et al. 2015). However, there is no consensus on what aspect of motor adaptation this decrease may relate to. Motor adaptation results from several interacting learning mechanisms, including implicit adaptation and strategy‐based learning. The implicit adaptation system is most sensitive to the presentation of visual feedback around 160 ms after movement onset, possibly corresponding to the time point of sensory prediction error computation (Wang et al. 2024). Here, movement duration was 310 ms on average. Thus, we would expect that neural processing related to implicit adaptation starts before movement offset. The PMBR, however, is a signal that follows movement offset, when the peak sensitivity of the implicit adaptation system to error is already over. Thus, a close association between implicit adaptation and PMBR amplitude seems a priori unlikely. On the other hand, the difference between desired and actual movement outcomes, which is the driving error for strategy‐based adaptation (Taylor et al. 2014; Taylor and Ivry 2011), is present upon movement offset, compatible with the timing of the PMBR.

Here, we contrasted two conditions that engaged strategy‐based motor learning to a different degree. In the 1× condition, motor adaptation was restricted to implicit learning by limiting visuomotor rotation to a single movement. In the 2× condition, adaptation resulted from a combination of implicit and strategy‐based learning. We observed a bias in the direction of hand movements after the introduction of the rotation in both conditions. After‐effects were bigger in the 2× condition, consistent with one additional rotated reach in the 2× condition, allowing further implicit adaptation. There were no differences in after‐effects in response to different rotation magnitudes, which is in line with the observation that implicit learning saturates for large rotations (Kim et al. 2018). Participants reaimed on the second rotated trial, with the movement angle depending on the magnitude of the visuomotor rotation, reflecting adjustment of a movement strategy. In agreement with increased cognitive load during reaiming (McDougle and Taylor 2019), RTs increased in the 2× condition, but not in the 1× condition.

We observed a strong decrease in PMBR amplitude after a visuomotor perturbation was introduced in both conditions. This decrease was more pronounced when participants could anticipate the same visuomotor rotation in the subsequent trial and could, therefore, reaim. Implicit learning is not sensitive to knowledge of future perturbation (Avraham et al. 2020). Any neural correlate strictly related to implicit learning should thus also be invariant to expectation.

However, strategy‐based adaptation interacts with implicit adaptation (Miyamoto et al. 2020; Tsay, Haith, et al. 2022; for review see Therrien and Wong 2022), and the extent of implicit adaptation may, therefore, not have been exactly identical in both conditions. Could an interaction between both learning mechanisms explain the stronger PMBR decrease in the 2× condition? The observed pattern of PMBR differences across trial types and conditions speaks against an association between any varying degrees of implicit adaptation in the two conditions and PMBR. Strategy‐based learning typically attenuates implicit adaptation (Albert et al. 2022; Benson et al. 2011). If the observed decrease in PMBR amplitude upon introduction of a visuomotor rotation was related to implicit adaptation, we would, therefore, expect a less pronounced reduction in PMBR in the 2× condition, when strategy use should diminish implicit adaptation. However, we observed the opposite, that is, a more pronounced reduction in PMBR amplitude. Together, our results indicate that PMBR is likely unrelated to implicit motor adaptation. The PMBR decrease in the 1× condition also speaks against the notion that PMBR decrease reflects strategy‐based learning per se, but instead a more general process. Error sensitivity (Tan, Jenkinson, et al. 2014) and timing of PMBR suggest a role in evaluation of movement error.

This interpretation is in line with the view that PMBR is signaling the saliency of movement errors (Torrecillos et al. 2015). Importantly, unlike in Torrecillos et al., saliency in our task was defined by the task goal, that is, by a priori knowledge about task relevance, rather than merely by deviance in sensory stimulation, which was constant across the 1× condition and 2× condition. We show that the PMBR decrease was amplified if participants used a movement strategy on the next trial. Importantly, movement kinematics and visual feedback of the movement were matched across conditions, at least in the critical trials (pre‐trial and first rotation). Thus, any differences in PMBR between conditions necessarily relate to the movement context and its relevance for future behavior. While the target error in the first rotated trial was relevant for the task in the 2× condition, that is, changing movement direction on the second rotated trial, it was task irrelevant on the next (unrotated) trial of the 1× condition, as the movement direction did not change. We conclude that PMBR may be modulated by the task relevance of errors.

Understanding the link between PMBR and error detection is essential for clarifying the role of reduced PMBR in clinical disorders—such as PD (Pfurtscheller et al. 1998; Tamás et al. 2003) and CD (Aoh et al. 2019; Visani et al. 2020)—which have been associated with deficits in motor adaptation. In patients with PD, reduced motor adaptation (Contreras‐Vidal and Buch 2003; Singh et al. 2019) is likely linked to impairments in strategic reaiming (Tsay, Najafi, et al. 2022). Moreover, the cerebral cortex, basal ganglia, and cerebellum form a highly interconnected network (Hintzen et al. 2018; Milardi et al. 2019). Neurodegeneration in spinocerebellar ataxia may also affect the basal ganglia, which play a key role in generating beta oscillations (Chikermane et al. 2024), potentially manifesting as reduced PMBR. Notably, patients with CD also exhibit impairments in strategic reaiming (Butcher et al. 2017; Wong et al. 2019). If a decrease in PMBR signals the detection of a behaviorally relevant perturbation, then a chronically reduced PMBR in these conditions, even during unperturbed movements, could impair the ability to notice and respond to task‐relevant changes, thereby contributing to the observed adaptation deficits. Based on this consideration and our prior finding that PMBR decrease precedes reaiming, we hypothesized that a drop in PMBR may reflect error detection and thus predict individual reaiming success. However, results from our LMMs did not support this hypothesis. A possible reason for this could be that the visuomotor rotations used in the present study, as well as our previous study (Korka et al. 2023), were large and, thus, easily detectable.

Strategy‐based adaptation likely unfolds in several consecutive steps. First, the target error of a given movement is registered and evaluated. This likely happens following the end of a movement, when error information is available that can guide future action. Depending on the movement context, this guidance may happen at a later stage, when error information is recalled from working memory (Hillman et al. 2024) and helps reduce future target error (Haith et al. 2015). There may be, therefore, a time gap between the first processing of an error and the recall of that error for imminent action. Indeed, in our experiment, the next movement towards the target was only initiated after returning to the starting position. Thus, PMBR likely does not reflect action selection for the immediate next movement, which was the return movement to the starting position, but rather the processing of movement errors for a more flexible update of a movement later in the future.

Reaiming accuracy, on the other hand, reflects both stages of developing a cognitive strategy—the evaluation of error and the updating of a motor plan. It may, therefore, not be surprising that the PMBR, that is, an EEG signal that follows past movement rather than preceding imminent movement, does not predict behavioral performance in strategic reaiming. Changes in premovement beta power have been associated with motor adaptation (Darch et al. 2020), with a stronger decrease over medial‐frontal areas related to strategic reaiming (Jahani et al. 2020). We therefore conducted a post hoc analysis of premovement beta desynchronization. Compared to the first rotated trial, the second rotated trial—when participants were preparing a reaiming movement—showed earlier and stronger beta desynchronization, potentially reflecting greater movement‐planning demands. This interpretation aligns with the observed increase in reaction time on the second rotated trial, suggesting heightened cognitive effort. Examining the relationship between reaiming performance and premovement beta power revealed that a larger decrease in premovement beta predicted a greater reduction in movement error during reaiming, even after accounting for RT. Notably, beta desynchronization on reaiming trials began at least 800 ms before movement onset, compared to ~400 ms on the preceding trial, whereas the average RT increase was only ~50 ms. This temporal difference cannot be explained by slower responses alone, suggesting that reduced premovement beta power reflects not just prolonged movement preparation but a general change in the planning process. Potential planning‐related mechanisms for beta desynchronization over central and parietal regions include motor imagery (Nam et al. 2011; Yuan et al. 2010) and mental rotation (Hongzhou H. Chen et al. 2014; Sasaoka et al. 2014), which may be engaged to simulate possible action outcomes and select the most effective one (McDougle and Taylor 2019).

Our interpretation that the modulation of PMBR codes for task‐relevance of error falls well in the status quo‐framework for cortical beta‐band activity, which posits that beta activity is “related to the maintenance of the current sensorimotor or cognitive state” (Engel and Fries 2010). Building on this framework, one interpretation is that task‐relevant errors signal changes in visuomotor contingencies, prompting trial‐by‐trial behavioral adjustments. However, this view is closely tied to the discrete, trial‐based structure typical of motor adaptation experiments. Here, we propose an alternative perspective that emphasizes the potential ecological relevance of PMBR in more continuous, real‐world settings. In the status‐quo framework, beta activity is attributed to an inhibitory role in the motor system. This aligns with findings that beta desynchronization during movement enhances cortico‐spinal excitability, whereas PMBR reduces cortico‐spinal excitability (R. Chen et al. 1998; Rhodes et al. 2024). Moreover, enhancing beta activity through transcranial alternating current stimulation has been shown to decrease movement speed (Pogosyan et al. 2009). Finally, successful stopping of initiated movements in a Go/NoGo task is associated with an increase in beta activity (Swann et al. 2009). Therefore, we speculate that a reduction in PMBR following the detection of task‐relevant movement errors may alleviate motor system inhibition, enabling immediate corrective movements and thus contributing to task success.

### 4.2 Limitations

PMBR has been associated with error saliency and error detection (Tan, Jenkinson, et al. 2014; Torrecillos et al. 2015). In the present study, we aimed to investigate whether PMBR correlates with reaiming accuracy. However, we did not observe such a relationship. Previous research suggests that participants can reliably detect visuomotor perturbations exceeding approximately 1.5 times their own motor variability (Gaffin‐Cahn et al. 2019). In our experiment, participants' motor variability during unperturbed trials was approximately 4°, while the visuomotor rotation applied was at least 30°, making the perturbations highly salient and easily detectable. We speculate that to uncover a link between individual PMBR and error detection, a broader range of visuomotor rotations—including smaller, less detectable perturbations—may be necessary.

Reaiming strategies can be developed by mentally rotating the aim point for a movement in the opposite direction of the target error by an appropriate angle. Depending on working memory capacity, established strategies can be reinstantiated by caching successful responses from memory (McDougle and Taylor 2019). We aimed to promote mental rotation and minimize response caching by increasing memory load, that is, use of three different visuomotor rotation magnitudes in two directions, respectively. Considering the number of repetitions and use of a single target, we cannot exclude the possibility that participants started caching responses over the course of the experiment. While this does not affect our interpretation of PMBR modulation by the task relevance of movement error—since both caching and reaiming rely on the same error signal—it remains unclear how these processes influence premovement beta power. Caching is associated with shorter RTs compared to mental rotation (McDougle and Taylor 2019), which could impact our estimate of premovement beta, as it was computed by averaging across a fixed time window. This may have led to an underestimation of the true relationship between premovement beta and reaiming performance, potentially explaining the small slope observed in our LMM.

Is it possible that the modulation of premovement beta did not result from reaiming itself but from deviating from the usual movement direction, that is the target direction? While movement direction can be inferred from M/EEG activity during movement, direction‐relevant information is encoded in low frequency activity and not in the beta band (Waldert et al. 2008). Indeed, beta activity is invariant across various aspects of movement (Kilavik et al. 2013) and thus likely is not affected by movement direction.

One aim of this study was to determine the effect of different motor adaptation mechanisms on PMBR. As mentioned above, we compare two experimental conditions that engage implicit adaptation alone or implicit and strategy‐based adaptation together. Thus, the effect of strategy‐based adaptation on PMBR cannot be observed in isolation. At a behavioral level, both learning mechanisms can be separated by selecting appropriate feedback presentation, for example, using movement invariant feedback to isolate implicit adaptation (Morehead, Taylor, et al. 2016), or delayed feedback to isolate strategy‐based adaptation (Brudner et al. 2016). However, such experimental manipulations of feedback introduce differences between conditions that, by themselves, may substantially affect neural correlates of movement.

### 4.3 Summary

We replicated our previous finding that strategy‐based motor adaptation reduces PMBR amplitude. Notably, this modulation was evident even on the trial preceding the execution of a movement strategy, suggesting a link to the detection of task‐relevant movement errors. However, we were unable to directly associate PMBR amplitude with interindividual differences in strategic reaiming accuracy, likely due to the high saliency of the error. Instead, premovement beta power emerged as a predictor of reaiming performance.

In conclusion, strategy‐based motor adaptation is a complex, multicomponent process involving both the detection of task‐relevant movement errors—reflected by PMBR—and the updating of movement plans—reflected by premovement beta power.

## Author Contributions

**Matthias Will:** conceptualization, data curation, formal analysis, funding acquisition, investigation, methodology, validation, visualization, writing – original draft, writing – review and editing. **Betina Korka:** conceptualization, data curation, formal analysis, methodology, writing – review and editing. **Max‐Philipp Stenner:** conceptualization, formal analysis, funding acquisition, methodology, project administration, resources, supervision, validation, writing – review and editing.

## Conflicts of Interest

The authors declare no conflicts of interest.

## Peer Review

The peer review history for this article is available at https://www.webofscience.com/api/gateway/wos/peer‐review/10.1111/ejn.70260.

## Supporting information


**Figure S1:** showing beta power locked to movement offset across different trial types and conditions.

## Data Availability

Kinematic raw data is available under 10.5281/zenodo.14671785. EEG raw data is available under 10.5281/zenodo.14673450 and 10.5281/zenodo.14674428.
