# Supplementary figures and images for: Involvement of Perimovement Neural Beta‐Oscillations in Strategic Aiming for Motor Adaptation
Source: Eur J Neurosci. 2025 Sep 26;62(6):e70260. doi: 10.1111/ejn.70260 (PMC12464804; doi:10.1111/ejn.70260)

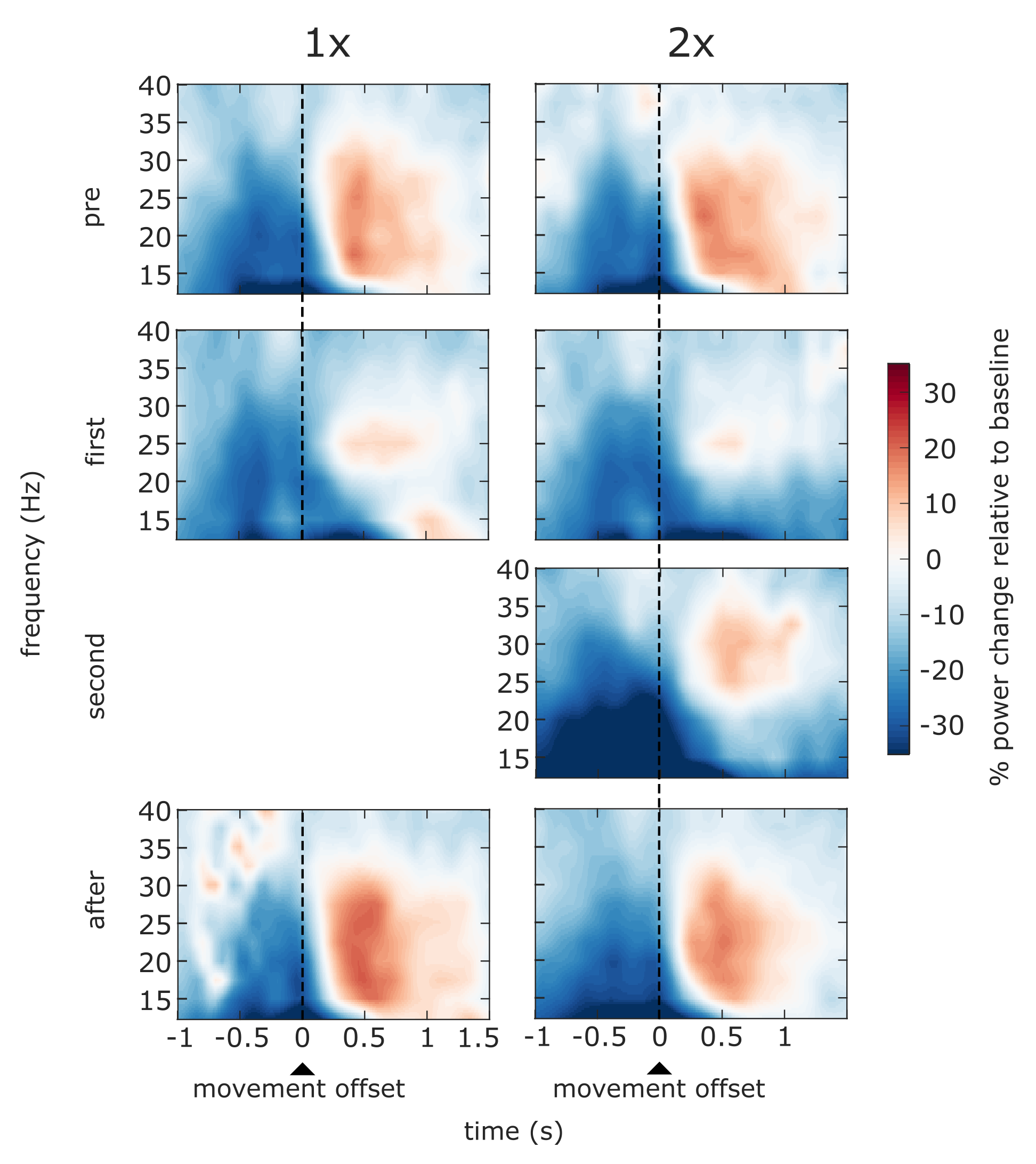

Supplement: Supplementary file 1 — Figure S1: showing beta power locked to movement offset across different trial types and conditions. [file EJN-62-0-s001.png]
